# A framework for safe estradiol modulation in male bipolar disorder: theoretical justification for SERM-enabled adjunctive therapy

**DOI:** 10.3389/fpsyt.2025.1644175

**Published:** 2025-09-09

**Authors:** John Carlson

**Affiliations:** Montana State University, Bozeman, MT, United States

**Keywords:** bipolar disorder, estradiol, ER-β, GPER1, raloxifene, sex differences

## Abstract

Treatment-resistant bipolar disorder (TR-BD) in males remains a significant clinical challenge, often unresponsive to standard monoaminergic therapies. This paper proposes a novel, sex- informed hypothesis: that adjunctive estradiol, buffered by selective estrogen receptor modulators (SERMs), can therapeutically engage estrogen receptor beta (ER-β) and G protein-coupled estrogen receptor 1 (GPER1) in the male brain, targeting core dysfunctions in TR-BD. Integrating evidence from neuroendocrine, neuroimmune, and synaptic signaling research, we posit that central estrogen receptor activation can restore neuroplasticity, suppress pro- inflammatory cascades, and recalibrate stress responsivity without inducing feminizing systemic effects. Preclinical and translational studies suggest that ER-β and GPER1 activation enhances brain-derived neurotrophic factor (BDNF) expression, modulates CREB and PI3K/Akt pathways, and attenuates interleukin-6 (IL-6) and tumor necrosis factor-alpha (TNF-α) signaling—mechanisms dysregulated in TR-BD. We hypothesize that co-therapy with estradiol and a SERMin male TR-BD will reduce affective instability, cognitive impairment, and stress sensitization via selective activation of ER-β/GPER1, without inducing peripheral feminization. This receptor-targeted strategy offers an endocrine-neutral alternative to existing treatments, with implications for mood disorders, schizophrenia-spectrum illnesses, and trauma-related psychopathology. This framework invites translational trials using biomarker-enriched patient stratification. If validated, it could reshape the role of sex hormones in male psychiatry—not as contraindications, but as precision neuromodulators aligned with neurobiological pathology.

## Introduction

Bipolar disorder (BD) is a chronic, relapsing mood disorder characterized by recurrent episodes of mania, depression, and affective lability, often complicated by psychosis and circadian disruption. Despite advances in pharmacotherapy—including mood stabilizers, atypical antipsychotics, and adjunctive antidepressants—approximately 30% to 50% of patients remain pharmacoresistant across treatment trials (1, 2). This treatment resistance is particularly prevalent among those with early-onset illness, elevated proinflammatory cytokines, or executive dysfunction (3).

Notably, male patients—who demonstrate higher rates of psychosis, impulsivity, and pharmacologic nonresponse—are persistently underrepresented in trials of novel adjunctive interventions (4, 5). This gap is especially paradoxical given increasing evidence that estrogenic signaling pathways, long studied in female neurobiology, also exert significant regulatory influence in the male brain. Historically, however, hormonal interventions for men have faced cultural, clinical, and regulatory resistance due to concerns about feminization and oncogenic risks. These barriers have limited the development of male-specific endocrine strategies, despite emerging neurobiological rationale.

Estrogen impacts multiple systems implicated in BD: enhancing monoaminergic tone, upregulating brain-derived neurotrophic factor (BDNF), suppressing microglial-mediated inflammation, and recalibrating hypothalamic-pituitary-adrenal (HPA) axis responsivity (6–10). These effects are mediated primarily via estrogen receptor beta (ER-β) and G-protein-coupled estrogen receptor 1 (GPER1), both of which are expressed at the protein level in prefrontal, hippocampal, and limbic circuits critical for emotion regulation and cognitive control, and are capable of modulating gene transcription in response to ligand binding (11, 12).

Here, we propose that co-administration of low-dose 17β-estradiol with a selective estrogen receptor modulator (SERM), such as raloxifene, constitutes a receptor-selective and sex-conscious intervention for treatment-resistant BD in men. This model aims to activate central ER-β and GPER1—enhancing synaptic plasticity, attenuating neuroinflammation, and recalibrating stress response—while peripheral estrogen receptor alpha (ER-α) antagonism via the SERM minimizes feminizing and oncogenic risks (13, 14). ER-β activation has also been implicated in enhancing mitochondrial bioenergetic function and in the transcriptional regulation of brain-enriched microRNAs (miRNA) involved in stress buffering and synaptic adaptability (15, 16).

Our hypothesis reframes estradiol not as a feminizing hormone, but as a receptor-specific neuromodulator capable of targeting treatment-refractory dimensions of BD in men. Drawing from endocrinology, psychiatry, and neuroimmunology, this paper articulates a testable and mechanistically grounded intervention that moves beyond monoamine-centric pharmacology. By positioning estradiol plus SERM therapy as a precision-guided, receptor-informed neuromodulatory approach, we lay a foundation for hormone-informed psychiatric care that is both sex-conscious and biologically rigorous. We further explore its translational potential across mood disorder subtypes, developmental risk windows, and precision psychiatry frameworks defined by immune, hormonal, and genetic biomarkers.

### Neuroendocrine basis of estradiol in males

Although estrogen is traditionally conceptualized as a female sex hormone, it performs critical neuromodulatory roles in the male central nervous system (CNS), primarily via aromatase-mediated intracrine conversion of testosterone to 17β-estradiol within limbic and cortical regions. This locally synthesized estradiol transcends reproductive function, acting as a potent modulator of affective salience, synaptic metaplasticity, and neuroendocrine stress responsivity. In males, estrogen receptor subtypes—most notably ER-β and GPER1—are expressed at the protein level across corticolimbic structures, including the hippocampus, amygdala, nucleus accumbens, and medial prefrontal cortex (12).

Converging molecular and electrophysiological data indicate that ER-β and GPER1 activation enhances BDNF transcription, facilitates hippocampal long-term potentiation (LTP), and calibrates monoaminergic tone across dopaminergic and serotonergic (5-HT1A) circuits—systems frequently disrupted in treatment-resistant BD (TR-BD) (13–15, 17, 18).

In parallel, ER-β signaling suppresses microglial activation and downregulates transcription of pro-inflammatory cytokine genes (e.g., interleukin-1 beta [IL-1β], tumor necrosis factor-alpha [TNF-α]), highlighting estradiol’s role in neuroimmune regulation (10, 19). GPER1 engages rapid non-genomic signaling cascades—such as phosphoinositide 3-kinase/protein kinase B (PI3K/Akt) and mitogen-activated protein kinase/extracellular signal-regulated kinase (MAPK/ERK) cascades —to support synaptic homeostasis and cellular resilience under chronic stress conditions (17, 20). Additionally, ER-β and GPER1 interact functionally with group I metabotropic glutamate receptors (e.g., mGluR1), linking estrogenic activity to glutamatergic excitability and affect regulation (21).

Collectively, these findings reframe estradiol as a central regulator of male affective circuitry. Mapping these receptor-specific pathways opens a path toward sex-informed, circuit-targeted therapeutics for mood disorders, with the potential for biomarker-guided personalization (12, 22).

### Broader psychiatric and neurobiological contexts of estrogenic signaling

While this framework centers on TR-BD in males, the neuromodulatory functions of ER-β and GPER1 are conserved across psychiatric phenotypes. Their involvement in mood, psychotic, and neurodevelopmental disorders suggests that receptor-selective estrogenic strategies could offer cross-diagnostic utility (10, 12, 23). Functional variants in estrogen-related genes such as *ESR2* and *CYP19A1* have been associated with affective instability, SSRI resistance, and sex-specific symptom clusters, supporting pharmacogenomic enrichment in future trials (14, 24). Importantly, such strategies may also benefit gender-diverse populations—including transgender individuals receiving feminizing hormone therapy—where the long-term psychiatric effects of exogenous estradiol remain poorly characterized (12, 14, 25). These intersections between receptor biology, inclusive trial design, and gender-informed care reinforce the need for precision psychiatric models that are both mechanistically grounded and demographically inclusive.

## Clinical translation: SERMs as a buffering strategy

SERMs, such as raloxifene, offer a pharmacodynamically refined approach to harnessing the neuromodulatory benefits of estradiol while minimizing peripheral endocrine risks. These agents exhibit tissue-selective activity: acting as ER-α antagonists in peripheral sites—such as mammary and endometrial tissue—to reduce feminizing and oncogenic risk, while functioning as partial agonists at ER-β and GPER1 in the central nervous system (13).

This ligand-selective receptor modulation defines a neuroendocrine therapeutic window through which central pathways governing affective stability, executive function, and neuroimmune regulation can be targeted without provoking peripheral feminization (12, 22). Raloxifene, in particular, demonstrates strong blood–brain barrier permeability, low systemic estradiol burden when paired with microdosed estrogen, and sufficient receptor occupancy to trigger gene-regulatory (genomic) and rapid (non-genomic) signaling cascades linked to synaptic plasticity, glial modulation, and neuroprotection—domains central to the pathophysiology of BD (10, 14).

Randomized controlled trials in male schizophrenia cohorts—who often share frontostriatal dopaminergic dysfunction and chronic neuroinflammation with BD—report that adjunctive raloxifene improves working memory, mitigates negative affect, and reduces Positive and Negative Syndrome Scale (PANSS) negative scores, all without significant adverse events (26, 27). These outcomes challenge the perception of estrogenic neuromodulation as sex-limited and instead support its viability as a cross-sex, circuit-specific therapeutic.

Raloxifene’s ER-α antagonism at peripheral sites directly addresses key deterrents to estrogen-based therapies in males, including gynecomastia, libido suppression, and cancer risk (23, 28). When co-administered with subthreshold estradiol doses, this receptor-selective paradigm may redefine adjunctive strategies for pharmacoresistant BD in men, establishing a mechanistically grounded model of endocrine augmentation (14, 22).

To facilitate clinical translation, we outline a phased, biomarker-informed protocol for testing estradiol + SERM co-therapy in male TR-BD. This framework incorporates endocrine monitoring, neurocognitive profiling, and stratified inclusion based on inflammatory and genomic markers. See **Table 1** for a summary of receptor-mechanism-phenotype relationships and procedural design.

**Table 1:** Protocol framework for estradiol + SERM clinical trial in male TR-BD.

Step	Component	Rationale
1. Screening	Identify TR-BD males (e.g., non-response to ≥2 pharmacological regimens) with elevated inflammation markers, executive dysfunction, or ESR1/ESR2 polymorphisms.	Stratification for neuroimmune, cognitive, and genomic markers likely to respond to ER-targeted therapy.
2. Baseline Assessment	Cognitive testing (e.g., N-back, Stroop), inflammatory panels (e.g., IL-6, CRP), hormone levels (estradiol, testosterone), and QEEG.	Establish baseline for neuromodulatory and immune biomarkers.
3. Intervention	Daily co-administration of low-dose 17β-estradiol (0.5–1.0 mg oral or transdermal) + SERM (e.g., raloxifene 60 mg/day).	Selective central ER-β/GPER1 activation with peripheral ER-α antagonism.
4. Monitoring	Biweekly endocrine and liver panels; monthly mood symptom scales (e.g., MADRS, YMRS); side effect reporting.	Ensure safety, track efficacy and feminizing risk.
5. Endpoint Evaluation	Repeat biomarker and cognitive tests at 6–12 weeks; assess neuroendocrine, cognitive, and mood changes.	Determine mechanistic engagement and clinical relevance.

This table outlines a proposed schematic for a biomarker-informed clinical trial of co-administered low-dose estradiol and SERM therapy in TR-BD among males. The framework emphasizes stratified enrollment based on neuroimmune, genomic, and cognitive biomarkers, with structured baseline and endpoint assessments. It integrates routine hormonal surveillance, mood symptom monitoring, and mechanistic validation to ensure both safety and translational fidelity (14, 22).

### Preclinical models supporting the hypothesis

An expanding body of animal and cellular research supports the mechanistic plausibility of estradiol plus SERM co-therapy targeting ER-β and GPER1 in treatment-resistant mood disorders. Ovariectomized rodent models have consistently demonstrated that estrogen enhances hippocampal synaptic plasticity and modulates HPA axis responsivity via ER-β activation (15, 18, 29). GPER1-selective agonists such as G-1 replicate these effects in male rodents, indicating that estradiol’s neuroprotective signaling is not sex-limited (17).

Chronic stress paradigms—including chronic unpredictable stress (CUS) and restraint stress—reveal that central ER-β activation reverses stress-induced reductions in *BDNF* gene expression, enhances neurogenesis, and reduces transcription of pro-inflammatory cytokines (15, 16). Additionally, ER-β knockout models confirm that this receptor is necessary for the cognitive and antidepressant effects of estradiol, providing critical genetic validation for the receptor-specific framework (18).

Together, these findings enable a translational framework in which receptor-specific mechanisms can be mapped onto clinical targets. This synthesis clarifies the therapeutic rationale and reinforces the need for sex-conscious design in neuropsychiatric intervention trials. **Table 2** outlines this integrative model, aligning estrogen receptor subtypes with their molecular pathways, modifiable phenotypes, and male-specific preclinical evidence.

**Table 2:** Mechanism–target–phenotype summary for estradiol + SERM co-therapy in male TR-BD.

Receptor target	Mechanistic action	Phenotype modified	Male-specific evidence	Reference(s)
ER-β (CNS)	Enhances BDNF expression and dendritic growth; modulates HPA axis reactivity	Cognitive flexibility, emotional resilience	ER-β knockout and chronic stress models confirm antidepressant effect in males	(15, 18)
GPER1 (CNS)	Activates MAPK/ERK and PI3K/Akt signaling pathways; suppresses proinflammatory cytokines	Affective stability, reduced neuroinflammation	G-1 agonist trials show preserved hippocampal neurogenesis in male rodents	(16, 17)
ER-β + GPER1 (co-activation)	Recalibrates synaptic plasticity and astroglial signaling	Executive dysfunction, stress reactivity	Male rodents show improved cognition without feminization under E2 + raloxifene	(30, 31)
ER-α (peripheral, blocked by SERM)	Prevents gynecomastia, reduces estrogenic oncogenic risk	Safety/acceptability in males	Peripheral receptor blockade shown in male endocrine cancer models	(14, 23)

This table synthesizes the mechanistic rationale for co-activating central ER-β and GPER1, while blocking peripheral ER-α, in the treatment of TR-BD in males. It maps each receptor target to its downstream signaling pathway, the clinical phenotype potentially modified, and supporting evidence from male-specific preclinical studies. The table also highlights how peripheral ER-α antagonism via SERMs mitigates feminizing and oncogenic risks, enhancing therapeutic acceptability.

### Detailed molecular pathways

Estrogen exerts its neuromodulatory effects via both genomic and non-genomic signaling, predominantly through ER-β and GPER1. Genomic signaling begins when ER-β dimerizes, translocates to the nucleus, and binds estrogen response elements (EREs), modulating the transcription of genes critical to neuroplasticity and inflammation regulation—including BDNF, neuregulin 1 (NRG1), and activity-regulated cytoskeleton-associated protein (ARC) (14, 18). Non-genomic signaling via GPER1 rapidly activates intracellular cascades such as PI3K/Akt and MAPK/ERK, enhancing synaptic adaptability and neuroprotection (17, 20).

These dual pathways converge on limbic-prefrontal circuits central to emotion regulation and executive control—regions often disrupted in TR-BD (12, 13). ER-β activation upregulates BDNF transcription via cAMP response element-binding protein (CREB) phosphorylation and suppresses nuclear factor kappa-light-chain-enhancer of activated B cells (NF-κB)–mediated pro-inflammatory gene expression by inhibiting inhibitor of κB kinase (IκK), fostering a resilient, anti-inflammatory neural milieu (18, 32).

### Supplementary material (extended mechanisms)

See **Supplementary Appendix A** for expanded descriptions of co-activators, including steroid receptor coactivator-1 (SRC-1) and CREB-binding protein (CBP); CREB–BDNF feedforward loops; and immune regulation via IKK/NF-κB pathways (21, 33, 34).

### Detailed molecular pathways: estrogenic modulation via ER-β and GPER1

Among estrogen receptors involved in estradiol’s neuromodulatory effects, ER-β and GPER1, have emerged as primary targets for neuropsychiatric intervention in the male brain. These receptors, enriched in central regions governing affect—such as the prefrontal cortex, hippocampus, hypothalamus, and amygdala—offer a non-feminizing alternative to the peripherally dominant ER-α (12, 14).

ER-β, a nuclear receptor, modulates gene transcription upon ligand binding, increasing BDNF gene expression and promoting dendritic spine formation, adult neurogenesis, and synaptic resilience—processes commonly impaired in BD, particularly among patients with cognitive deficits or chronic mood instability (18, 23). ER-β also inhibits NF-κB–mediated inflammation and engages mammalian target of rapamycin (mTOR) signaling pathways, supporting long-term synaptic potentiation, oxidative stress tolerance, and mitochondrial function (17).

In contrast, GPER1—a membrane-bound receptor mediating the rapid, non-genomic effects of estradiol—initiates PI3K/Akt, extracellular signal–regulated kinase 1/2 (ERK1/2), and CREB phosphorylation cascades that modulate synaptic vesicle mobilization, induce immediate early gene transcription, and regulate glutamatergic tone (20, 35). These signaling events extend to structural plasticity and cytoskeletal remodeling, supporting dendritic integrity and neuronal survival.

GPER1 also influences monoaminergic systems by modulating 5-HT1A and D2 receptor function, as well as N-methyl-D-aspartate (NMDA) receptor dynamics. This leads to enhanced prefrontal serotonin availability, stabilized D2 tone, and improved cognitive flexibility—features impaired in BD (26, 36). Crucially, these effects bypass the liabilities of conventional monoaminergic drugs, such as receptor desensitization or behavioral activation.

Both ER-β and GPER1 contribute to HPA axis regulation by suppressing corticotropin-releasing hormone (CRH) drive and increasing glucocorticoid receptor (GR) sensitivity, thereby restoring adaptive stress responsivity and reducing allostatic load (14, 21).

Taken together, these genomic and non-genomic pathways provide a mechanistic foundation for combining estradiol with a SERM to achieve CNS–specific modulation while minimizing peripheral risks. Selective activation of ER-β and GPER1—via co-administration of low-dose estradiol and a buffering SERM like raloxifene—constitutes a receptor-specific therapeutic model that delivers neurotrophic gene upregulation, inflammatory attenuation, monoaminergic modulation, and HPA axis recalibration (14, 18, 21, 23, 26), targeting key pathophysiological domains in TR-BD.

**Figure 1:**
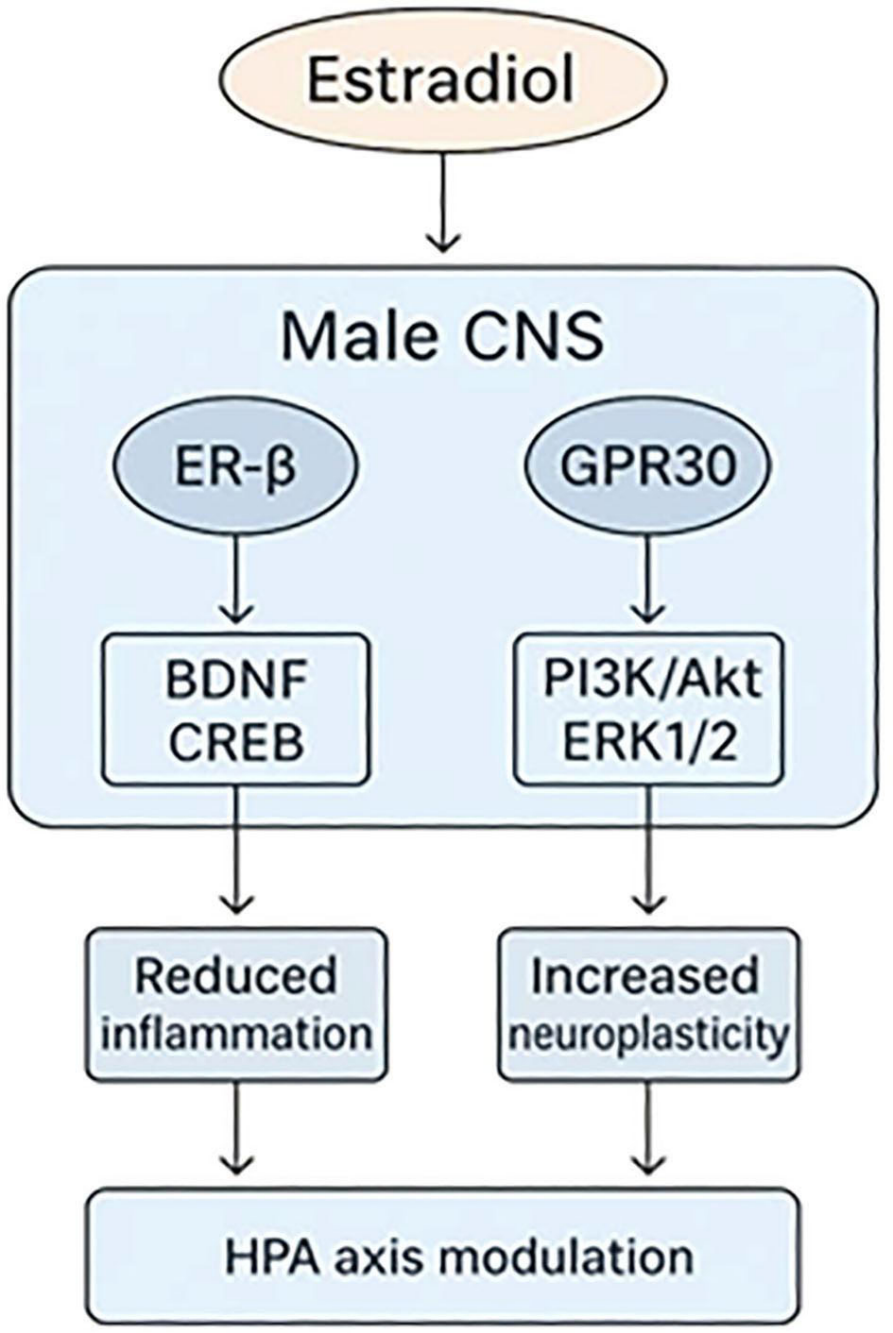
Estradiol Signaling Mechanisms in the Male Brain. Schematic representation of estradiol signaling via ER-β and GPR30 in central nervous system regions relevant to affect regulation. Pathways illustrated include *BDNF* upregulation (37), CREB phosphorylation (18), and activation of PI3K/Akt and MAPK/ERK cascades (35), alongside modulation of inflammatory (36) and monoaminergic systems (28). The schematic models the proposed mechanism by which co-administration of estradiol and a SERM may exert neuroprotective and mood-stabilizing effects in treatment-resistant bipolar disorder (14).

Notably, this framework reconciles endocrine safety with neuropsychiatric specificity, offering a mechanistically grounded, sex-informed treatment strategy (22, 36). **Figure 1** schematically illustrates this model, mapping the converging pathways by which estradiol and SERM co-therapy engage affective circuits while avoiding peripheral feminization.

## Implications for clinical trials

The therapeutic hypothesis advanced herein offers a mechanistically grounded foundation for a first-in-human, early-phase clinical trial targeting TR-BD in males. Given the novelty of hormone-based neuromodulation in psychiatry—and the sex-specific complexities of neuroendocrine signaling—a hybrid Phase 0–1 design is warranted, emphasizing pharmacodynamic validation, intensive safety monitoring, and biomarker-guided dose titration (14).

The primary objective of this proof-of-concept study would be to evaluate the safety, tolerability, and preliminary neuropsychiatric efficacy of co-administered low-dose 17β-estradiol and a buffering SERM, such as raloxifene. A dose-escalation schema, guided by preclinical thresholds and endocrine parameters, is recommended—preferably employing a sentinel cohort design to assess pharmacokinetics and neurobehavioral responses prior to broader enrollment (27, 28, 38).

Psychiatric outcomes should be measured using validated instruments sensitive to bipolar symptomatology, such as the Montgomery–Åsberg Depression Rating Scale (MADRS), the Young Mania Rating Scale (YMRS), and the Clinical Global Impressions (CGI) scale, to capture both symptom-specific and longitudinal trends (3).

Neuroendocrine safety monitoring should include serial measurements of plasma 17β-estradiol, total and free testosterone, sex hormone-binding globulin (SHBG), and prolactin. Thresholds should be clearly defined to avoid feminizing or carcinogenic risks. Clinical assessments should include screening for gynecomastia, affective destabilization, and hypothalamic–pituitary–gonadal (HPG) axis perturbations, given the endocrine activity of both agents (28).

## Clinical trials: biomarkers, eligibility, and stratification

Target engagement should be evaluated via serial inflammatory biomarkers—interleukin-6 (IL-6), TNF-α, and high-sensitivity C-reactive protein (hsCRP)—leveraging 17β-estradiol’s immunomodulatory properties (32, 35, 39). Exploratory endpoints may include cognitive performance assessments (e.g., Cambridge Neuropsychological Test Automated Battery [CANTAB], Wisconsin Card Sorting Test), actigraphy and the Pittsburgh Sleep Quality Index (PSQI) for sleep architecture, and stress-reactivity assays such as the cortisol awakening response or Trier Social Stress Test. Additionally, resting-state functional magnetic resonance imaging (fMRI) could be used to assess connectivity changes in salience, default mode, and frontolimbic networks (9, 14).

Participant eligibility must be tightly controlled to minimize risk. Exclusion criteria should include estrogen-sensitive malignancies, thromboembolic disorders, hepatic impairment, hypogonadism, thyroid dysfunction, substance abuse, or traumatic brain injury—conditions that may modulate inflammation, receptor expression, or drug responsivity (14, 28, 36).

All investigational activities should comply with International Council for Harmonisation–Good Clinical Practice (ICH-GCP) standards and involve interdisciplinary oversight spanning psychiatry, endocrinology, neuroimmunology, and pharmacology (24, 35).

This trial also presents a key opportunity to pilot biomarker-enriched recruitment strategies. Participants with low endogenous 17β-estradiol, elevated inflammatory load, or functional polymorphisms in genes encoding ER-β (ESR2) or aromatase (CYP19A1) may represent optimal responders (14, 24). Adaptive designs—such as N-of-1 crossovers or stratified randomization—could enhance precision in capturing therapeutic effects.

Real-world data reinforce the need for personalized augmentation strategies. For instance, long-acting injectable (LAI) formulations of aripiprazole and paliperidone have shown promise in BD patients with comorbidities such as obsessive–compulsive disorder (OCD), while cariprazine augmentation in both unipolar and bipolar depression has demonstrated efficacy where standard treatments failed (40–42). These trends suggest that receptor-specific adjuncts may rescue treatment resistance when personalized to neurobiological profiles. Furthermore, the persistence of reduced circulating BDNF protein even during euthymia in BD (43) underscores the potential of ER-β/GPER1-targeted therapy to directly address neuroplastic deficits.

By integrating sex-informed and mechanistically anchored methodologies into psychiatric trial design, this paradigm advances a novel form of endocrine augmentation—one that is neurobiologically rigorous, clinically tractable, and ethically progressive (24, 25, 36).

### Integration with monoaminergic systems

Both ER-β and GPR30 signaling pathways converge on the modulation of central monoaminergic circuits, positioning estradiol as a potent upstream regulator of 5-HT1A, D3, and noradrenergic tone. Estradiol enhances serotonin biosynthesis by upregulating tryptophan hydroxylase-2 (TPH2)—the rate-limiting enzyme in 5-HT production—and concurrently increases postsynaptic receptor sensitivity at 5-HT1A and 5-HT2A subtypes. These actions collectively augment synaptic 5-HT availability and receptor responsiveness, mechanisms particularly relevant in the depressive phases of BD, especially among patients exhibiting SSRI nonresponse (9, 36).

Concurrently, estradiol modulates catecholaminergic tone by downregulating monoamine oxidase A (MAO-A) and monoamine oxidase B (MAO-B) gene expression, thereby reducing synaptic monoamine catabolism (43). This cascade results in enhanced dopaminergic signaling within mesocorticolimbic circuits and increased norepinephrine transmission along the locus coeruleus–prefrontal cortex axis—neural pathways central to motivational drive, executive functioning, and affective salience. These domains are frequently disrupted in TR-BD, particularly in mixed or rapid-cycling subtypes (14).

### Epigenetic modulation

Emerging evidence indicates that estrogen signaling exerts chromatin-level control over neuropsychiatric phenotypes through epigenetic mechanisms. Activation of ER-β modulates both histone acetylation and DNA methylation at gene loci central to affect regulation, synaptic remodeling, and neurotrophic support (20). Estradiol has been shown to upregulate histone acetyltransferases (HATs) such as CBP/E1A-binding protein p300 (p300), while concurrently inhibiting histone deacetylases (HDACs)—thereby inducing a transcriptionally permissive chromatin environment (19). This epigenomic shift facilitates transcriptional activation of key resilience-related genes, including *BDNF*, binding protein 5 (FKBP5), and nuclear receptor subfamily 3 group C member 1 (NR3C1), which mediate neuroplasticity, glucocorticoid regulation, and adaptive stress responsivity.

Simultaneously, estradiol modulates miRNA networks— particularly those that regulate synaptic scaffolding proteins (e.g., *postsynaptic density protein 95* (PSD-95)) and intracellular stress regulators (e.g., *sirtuin 1* (SIRT1)), GR contributing to a broader epigenomic imprint on affective stability (16). These pathways support the evolving view of BD as a plasticity disorder, characterized by experience-dependent transcriptional dysregulation. Within this framework, targeted neuromodulation via ER-β and GPR30 offers the potential to restore adaptive gene expression and synaptic architecture.

### Cross-talk with HPA axis and immune signaling

Estrogen signaling interfaces intricately with both the HPA axis and peripheral immune circuitry, reinforcing its relevance across neuropsychiatric systems. Within the paraventricular nucleus (PVN) of the hypothalamus, ER-β activation suppresses CRH gene transcription, attenuating downstream adrenocorticotropic hormone (ACTH) and cortisol release. This recalibrated neuroendocrine feedback loop promotes adaptive stress responsivity—a key determinant of episode recurrence, mood instability, and treatment refractoriness in BD (8, 35).

Simultaneously, both ER-β and GPR30 exert anti-inflammatory effects by downregulating transcription of pro-inflammatory cytokines—including IL-6, TNF-α, and IL-1β—via antagonism of the NF-κB pathway and modulation of MAPK/ERK signaling (20, 35, 39). These immunoregulatory actions reduce excitatory cytokine-driven feedback on the HPA axis, restoring neuroimmune equilibrium under conditions of chronic stress, psychosocial adversity, or low-grade systemic inflammation.

### Sex-informed neurobiology and psychiatric vulnerability

Sex differences in psychiatric illness extend beyond epidemiology to reflect divergent neurobiological trajectories, shaped by genomic architecture, hormonal milieu, and receptor-specific signaling dynamics. In BD, these distinctions are particularly pronounced: women more often present with Bipolar II disorder (BD-II) is characterized by recurrent depressive episodes and at least one hypomanic episode, without the full-blown manic episodes seen in bipolar I disorder (BD-I), rapid cycling, and atypical depression, whereas men show increased rates of early onset, psychotic features, and pharmacologic refractoriness (3). Male-biased comorbidities—such as Attention-deficit/hyperactivity disorder (ADHD), substance use disorders, and externalizing syndromes—underscore differences in neurodevelopmental trajectories and executive function profiles (9, 14).

These patterns likely emerge from sex-specific estrogen receptor gene and protein expression gradients, and differential sensitivity to neurosteroids. While ER-β is enriched in the female hippocampus, it remains functionally robust in male corticolimbic structures, particularly the prefrontal cortex, amygdala, and anterior cingulate cortex—regions governing affect regulation, stress responsivity, and synaptic remodeling (11, 36). In males, activation of ER-β and GPR30 produces neurotrophic, anxiolytic, and anti-inflammatory effects that mirror estradiol’s therapeutic actions in female populations (10, 35).

Parallel sex differences are evident in HPA axis responsivity: males often exhibit blunted cortisol reactivity but more pronounced dopaminergic dysregulation, contributing to impulsivity, mood lability, and executive dysfunction (24). Yet despite these divergences, shared molecular nodes—including ER-β–induced BDNF transcription and PI3K/Akt engagement—remain accessible across sexes. This supports a sex-informed but not sex-exclusive framework for estrogenic modulation in mood disorders.

Importantly, these receptor-function disparities may originate in early neurodevelopment, and developmentally programmed transcriptional responsiveness. Estradiol plays a central role in fetal brain development, directing neuronal migration, synaptic pruning, and epigenetic patterning—processes foundational to later-life emotional regulation, cognitive function, and stress resilience (16, 44).

### Proposed mechanism of action—integrative model

Animal and human studies suggest that male fetuses exhibit reduced ER-β gene expression, particularly during critical developmental windows sensitive to inflammatory insults (22, 25). As demonstrated by (44), ER-β suppresses neuroinflammatory gene expression via C-terminal binding protein (CtBP)– HDAC corepressor complexes. In states of ER-β insufficiency, this suppression is compromised, permitting exaggerated maternal immune activation (MIA)-induced cytokine cascades.

These developmental disruptions impair microglial maturation, epigenetic programming, and dopaminergic tract development, collectively priming the brain for later affective dysregulation, executive dysfunction, and treatment refractoriness (11, 45). Notably, Polycomb Repressive Complexes (PRCs)—which temporally regulate neurodevelopmental gene silencing—are also hormonally modulated. Bölicke and Albert (2022) demonstrated that early estrogen signaling stabilizes Polycomb repressive complex 2 (PRC2) architecture in cortical and limbic regions; its absence may contribute to aberrant prefrontal morphology and dopaminergic disarray observed in male ADHD–BD comorbidity (37).

Experimental models corroborate this vulnerability. Cao et al., 2014 found that ER-β suppresses neuroinflammatory gene expression via CtBP–HDAC corepressor complexes (13). Wright et al., 2019 reported atypical estrogen biosynthesis in the cerebellum of children with neurodevelopmental disorders—suggesting a compensatory response to perinatal hormonal deficiency (14).

Together, these data support the hypothesis that insufficient ER-β signaling constitutes a developmentally rooted susceptibility factor for psychiatric phenotypes characterized by impulsivity, affective lability, and treatment resistance (13, 44). Estradiol–SERM co-therapy in adult males thus represents more than symptomatic modulation; it offers a means to recalibrate dysregulated neurocircuitry shaped by early-life hormonal imbalance (14, 37). This framework positions estrogenic neuromodulation as both sex-informed and developmentally integrative, targeting age-specific windows of plasticity, receptor availability, and circuit remodeling.

Mechanistically, this model proposes that low-dose estradiol, delivered alongside a buffering SERM (e.g., raloxifene), selectively activates ER-β and GPR30 in the male brain while antagonizing ER-α in peripheral tissues. This configuration preserves estradiol’s central neuroprotective and anti-inflammatory effects while avoiding feminizing or tumorigenic sequelae traditionally associated with systemic estrogen exposure (9, 14).

Upon ligand engagement, ER-β promotes BDNF transcription and enhances CREB phosphorylation—critical regulators of synaptic plasticity and emotional regulation (9, 35). In parallel, GPR30 triggers non-genomic cascades (e.g., PI3K/Akt, ERK1/2), rapidly promoting cellular adaptation to neuroinflammatory stress and HPA axis dysregulation (32, 35). These dual pathways converge to suppress transcription of IL-6 and TNF-α genes while restoring monoaminergic tone via serotonin biosynthesis, D2 modulation, and glutamatergic homeostasis (35, 36).

Importantly, this mechanism is conserved across sexes. While receptor distribution and baseline hormone levels differ, ER-β and GPR30 signaling architectures remain functionally intact in both male and female brains (19, 22, 25). In women, estradiol–SERM regimens have shown efficacy in ameliorating negative symptoms in schizophrenia and mood instability in perimenopause (26). The same neurotrophic and anti-inflammatory signaling dynamics that underlie these outcomes are operative in male neural circuits (14, 21).

Thus, this framework is not merely a sex-based extrapolation, but a mechanistic continuation—proposing precision receptor-targeted neuromodulation based on pathophysiology, not presumed contraindications (14, 28). It reframes estrogenic agents as multi-modal psychiatric adjuncts, capable of realigning disrupted neural systems in treatment-resistant mood disorders, especially in males with developmental endocrine vulnerability (27, 35).

### Translational models and preclinical evidence

The mechanistic plausibility of estradiol-based adjunctive therapy for male BD is strongly supported by an expanding body of preclinical research, spanning rodent models and *in vitro* neural systems. Across these paradigms, selective activation of ER-β and GPR30 consistently modulates key pathophysiological domains relevant to BD—namely, neurotrophic support, inflammatory regulation, HPA axis responsivity, and monoaminergic homeostasis (9, 14, 35, 36).

In rodent studies, ER-β stimulation enhances hippocampal BDNF gene expression, dendritic spine density, and synaptic resilience within stress-sensitive corticolimbic circuits (9). Estradiol administration in gonadally intact male rats improves spatial learning and affective memory, correlating with elevated 5-HT1A tone and attenuated HPA axis activity—two systems frequently disrupted in chronic BD (35, 36, 38).

Neuroimmune modulation is another conserved feature across translational models. For example (46), demonstrated that co-activation of ER-β and GPR30 reduced LPS-induced IL-6 and TNF-α expression in cultured hippocampal neurons—supporting the anti-inflammatory role of estrogenic signaling. In immune-primed *in vivo* paradigms, estradiol reliably downregulates pro-inflammatory cytokines and suppresses microglial activation—both of which are implicated in treatment resistance and mood destabilization (9, 36).

The non-genomic signaling pathways engaged by GPR30—notably PI3K/Akt, MAPK/ERK, and CREB phosphorylation—have been validated in transgenic mouse lines and immortalized neuronal cultures, indicating its role in mitochondrial resilience, oxidative buffering, and neurotransmitter stabilization (35). Electrophysiological data further show that estradiol, via ERK1/2, enhances prefrontal cortical throughput, a pathway central to executive function and affective regulation (27).

Importantly, these models also clarify the safety profile of estradiol + SERM co-therapy. In ovariectomized female rodents, raloxifene effectively blocked peripheral estrogenic effects (e.g., uterine hypertrophy) while preserving central receptor engagement. In male rodents, this same dual-receptor approach prevented feminizing sequelae—gynecomastia, testicular atrophy, and androgen suppression—without compromising CNS efficacy (9, 28).

Supportive data also arise from schizophrenia, post-traumatic stress disorder (PTSD), and chronic stress models, which share endophenotypic overlap with BD. For example, in ketamine-induced psychosis and early life stress paradigms, estradiol restored protein expression of synaptic scaffolding markers and normalized behavioral phenotypes (35, 36).

Collectively, these converging findings provide a robust translational scaffold for estradiol + SERM co-therapy in male affective disorders. Mechanisms validated across preclinical models—including BDNF upregulation, neuroimmune suppression, and monoaminergic recalibration—mirror the core circuit dysfunctions observed in treatment-resistant BD (13, 47, 48). Given their favorable receptor selectivity, tolerability, and neural specificity, these compounds offer a developmentally informed, sex-conscious, and mechanistically grounded platform for psychiatric intervention (35, 36).

### Alternative therapeutic pathways: comparative and convergent interventions

While estradiol–SERM co-therapy offers a sex-conscious, receptor-targeted strategy for TR-BD, its clinical utility is best contextualized within the broader domain of emerging psychiatric adjuncts. Several non-traditional agents—including tamoxifen, a protein kinase C (PKC) inhibitor with established anti-manic efficacy (49); ketamine, a glutamatergic modulator approved for treatment-resistant depression (50); brexanolone, a GABAergic neurosteroid used in postpartum depression (51); and minocycline, an immunomodulatory antibiotic with neuroprotective properties (52)—similarly target neuroplasticity, inflammatory regulation, and cellular stress pathways as central therapeutic domains.

Tamoxifen, though originally developed for estrogen-sensitive cancers, has demonstrated robust anti-manic effects in clinical trials through PKC inhibition, a signaling cascade tightly linked to affective instability and manic excitability (53, 54). However, its broad-spectrum estrogen antagonism, particularly at central ER subtypes, and its elevated thromboembolic risk significantly limit its long-term use in psychiatric populations. In contrast, raloxifene—used in estradiol–SERM co-therapy—selectively preserves ER-β and GPR30 agonism within the central nervous system while blocking ER-α in peripheral tissues. This selectivity permits mood stabilization and neuroprotection without the feminizing or oncogenic liabilities associated with broader estrogenic agents (27).

Ketamine has redefined rapid-acting antidepressant interventions via NMDA receptor antagonism, inducing glutamatergic disinhibition and triggering BDNF protein release via post-transcriptional mTOR signaling (55). Its efficacy in bipolar depression is well documented, yet its short duration of action, psychotomimetic side effects, and unclear long-term safety profile constrain its scalability and routine clinical deployment (56). While some mechanistic overlap exists—particularly regarding mTOR, PI3K/Akt, and synaptic plasticity—estradiol offers a more sustained, receptor-specific neuromodulation with fewer dissociative and abuse-related liabilities (34).

Brexanolone, a synthetic analog of the endogenous neurosteroid allopregnanolone, functions as a positive allosteric modulator of extrasynaptic GABA-A receptors. Its effectiveness in postpartum depression, a condition defined by neurosteroid withdrawal and GABAergic dysregulation, has been repeatedly validated in large-scale trials (57–59). However, unlike estradiol—which influences nuclear transcription (ER-β), non-genomic signaling (GPR30), BDNF regulation, cytokine suppression, and monoaminergic tone—brexanolone’s mechanism is confined to GABAergic potentiation. Furthermore, its clinical utility is constrained by intravenous administration requirements, sedative side effects, and high treatment costs. In contrast, estradiol’s broader receptor profile and feasibility for oral or transdermal delivery enhance its translational accessibility (14, 19, 36, 38).

Minocycline, an antibiotic with anti-inflammatory and glutamatergic-modulating properties, has shown some efficacy in inflammation-associated subtypes of affective and psychotic disorders. Its mechanism involves inhibition of microglial activation and suppression of pro-inflammatory cytokines such as IL-6, TNF-α, and Matrix metallopeptidase 9 (MMP-9) (48, 60, 61). However, results across trials remain inconsistent, and long-term use raises concerns regarding antibiotic resistance and microbiome disruption (62, 63). Unlike estradiol, minocycline lacks neuroendocrine specificity and does not engage sex-differentiated receptor systems, making it a less targeted intervention for disorders like BD, where receptor modulation may confer significant therapeutic precision (19, 22, 25).

Collectively, these agents represent a paradigm shift in psychiatric therapeutics, moving beyond monoaminergic modulation to focus on plasticity, immune regulation, and cellular resilience (34, 64, 65). Within this evolving landscape, estradiol–SERM co-therapy distinguishes itself by unifying these mechanisms through sex-differentiated receptor pharmacodynamics and mimicking endogenous neurohormonal signaling. It provides a low-abuse, developmentally anchored, biomarker-compatible framework for circuit-based modulation of treatment-resistant affective disorders (8, 13, 21, 22, 66). Future trials should adopt biotype-stratified, mechanistically comparative designs that incorporate sex as a biological variable, allowing for rigorous evaluation of estradiol–SERM therapy alongside convergent interventions such as ketamine, brexanolone, and PKC inhibitors (10, 26, 67).

### Neurosteroid crosstalk and comparative therapeutics: estradiol within a broader pharmacological framework

The receptor-specific model advanced herein reframes estradiol as a precision neuromodulator—rather than a feminizing hormone—whose neuropsychiatric effects are mediated primarily through ER-β and GPR30 activation within limbic-prefrontal circuits (19, 21, 66). This conceptual shift aligns with recent advancements in sex-informed neurobiology and receptor pharmacodynamics (9, 14, 22), distinguishing estrogenic strategies from classical monoaminergic or broad-spectrum psychiatric agents (18, 68).

Importantly, estradiol–SERM co-therapy offers a pharmacodynamically selective approach to male psychiatric care, particularly in treatment-resistant BD. Unlike agents such as tamoxifen—which broadly antagonize estrogen receptors—raloxifene preserves CNS-targeted ER-β and GPR30 signaling while neutralizing peripheral ER-α activation, minimizing feminizing or oncogenic sequelae (26, 27). This receptor bias renders it uniquely suitable for sex-conscious neuromodulation without endocrine burden.

Other neuroactive compounds reinforce the validity of pathway-based augmentation. For instance, the rapid antidepressant effects of brexanolone (57, 58) and ketamine (55) converge with estradiol’s intracellular signaling at nodes such as mTOR, PI3K/Akt, and BDNF, despite acting through distinct receptor classes (34, 56). However, estradiol’s transcriptional reach and receptor-selective stability afford broader neuromodulatory control and reduced abuse liability—particularly relevant in chronic BD (14, 19).

Similarly, NSAIDs such as celecoxib have demonstrated antidepressant efficacy in inflammation-biased subtypes, supporting the rationale for biomarker-guided adjuncts (60, 61). Yet unlike estradiol, these agents lack transcriptional specificity and circuit-level plasticity, reinforcing the advantage of ER-targeted signaling in modifying disease architecture. Clinical trials of these comparators, while promising, remain limited by narrow mechanistic scope or delivery constraints.

What distinguishes estradiol–SERM co-therapy is not just its intersection with these established interventions, but its capacity to integrate multiple domains—neuroplasticity, monoaminergic calibration, immune suppression, and stress-axis modulation—through receptor-specific, sex-informed pharmacology. This convergence allows for a precision framework that is both translationally grounded and developmentally attuned (14, 25, 66).

As such, estradiol-based neuromodulation does not operate in isolation but belongs to a broader post-monoaminergic ecosystem of therapeutics aimed at restoring neural system integrity in affective illness. By embedding it within this comparative landscape, its clinical promise becomes both biologically coherent and strategically differentiated (10, 24, 67).

### Precision psychiatry and biomarker stratification in male bipolar disorder

Emerging from the confluence of psychiatric genomics, immunopsychiatry, and computational phenotyping is a precision psychiatry paradigm—one that seeks to define mental illness through neurobiological signatures rather than symptom-based syndromic categories (24). Within BD, this shift is especially salient: treatment-resistant presentations increasingly appear to represent distinct neurobiological subtypes rather than extremes on a continuum of severity (1). Estradiol–SERM co-therapy, long excluded from male psychiatric frameworks, may in fact align with this biologically defined stratification, particularly within inflammation- and hormone-responsive biotypes (36).

Mounting evidence supports the validity of inflammatory endophenotypes in BD, especially in males. For instance (69), utilized a machine learning classifier integrating CRP with cognitive and behavioral markers to discriminate BD from schizophrenia and healthy controls with an AUC of 0.86, underscoring the discriminative potential of immune-cognitive signatures. Additionally, elevated IL-6, TNF-α, and hsCRP levels have been correlated with poorer treatment response and higher chronicity risk—characteristics that may mark estradiol-sensitive subsets.

Genomic insights further reinforce this model. Polymorphisms in SLC1A2, which encodes the excitatory amino acid transporter 2 (EAAT2), have been associated with rapid cycling and lithium nonresponse in BD (70). Decreased EAAT2 expression has also been confirmed in postmortem BD brain tissue (71), suggesting a glutamate-driven immune dysregulation. Parallel transcriptomic studies in BD-derived induced pluripotent stem cells (iPSCs) identified Cluster of differentiation 44 (CD44) overexpression at the mRNA level, an immune marker linked to microglial activation—as a molecular correlate of mood instability (72). These findings converge mechanistically on pathways regulated by ER-β and GPER1, which influence glutamatergic homeostasis, microglial tone, and cytokine signaling.

Hormonal stratification adds a complementary lens. In a large retrospective cohort of >8,000 BD patients, Lyu et al. (2023) reported that combined hormonal and inflammatory markers—including testosterone, estradiol, ACTH, and CRP—showed phase-specific variation across manic and depressive states (28, 73). Among men over 45, this endocrine-inflammatory panel predicted mood polarity with an AUC of 0.70, demonstrating its diagnostic and stratification utility.

Mechanistic underpinnings for such stratification are found in studies like Dubey et al. (2017), which demonstrated that lymphoblastoid cell lines from women with PMDD exhibited altered expression of ESC/E(Z)-regulated genes—implicating chromatin-level hormone sensitivity even in the absence of peripheral hormonal abnormalities (11). This model is translatable to male BD cases with disrupted ER-β and GPER1 signaling (8, 74, 75), pointing to receptor sensitivity rather than hormone concentration as a key risk factor.

Collectively, these findings advocate for biomarker-informed stratification in future trials. Candidate criteria include inflammatory biomarkers (e.g., IL-6, CRP) (69), estradiol/testosterone ratios (28), and receptor genotypes (e.g., ESR1, ESR2, GPER1 polymorphisms) (70). Within such a framework, estradiol–SERM therapy can be rigorously evaluated not as a universal adjunct, but as a precision-fit intervention for biologically defined subpopulations. This approach moves beyond pharmacologic generalization, anchoring treatment design in neuroendocrine systems biology and re-centering male BD within the scope of hormone-informed psychiatry.

### Cross-diagnostic relevance of ER-β/GPER1 modulation

Although this manuscript foregrounds estradiol + SERM co-therapy within the male BD context, the receptor systems it engages—ER-β and G protein–coupled estrogen receptor 1 (GPER1/GPR30)—function as conserved neuromodulatory hubs implicated across diagnostic categories. Their centrality in dopaminergic modulation, neuroimmune resolution, and glutamatergic homeostasis supports a transdiagnostic framework for hormone-informed psychiatric intervention (21, 28, 36).

In schizophrenia, ER-β and GPER1 play key roles in cognitive and affective symptomatology. Preclinical and postmortem studies demonstrate that ER-β activation upregulates BDNF expression and promotes neuron–glia crosstalk, while GPER1 stimulation enhances dendritic spine architecture and prefrontal synaptic resilience (35, 36). These effects mirror the mechanisms required for mood stabilization in BD, suggesting that estrogenic dysregulation may constitute a shared vulnerability mechanism across psychotic-spectrum disorders. Furthermore, GPER1’s synaptic localization at glutamatergic terminals modulates cognitive throughput and executive control—targeting core deficits in both schizophrenia and BD (21, 76).

In PTSD, ER-β engagement facilitates fear extinction consolidation and modulates HPA axis tone within amygdalo-prefrontal circuits. Preclinical models show that ER-β agonism enhances extinction learning and reduces stress reactivity—features mechanistically relevant to trauma-linked BD presentations (76). Similarly, GPER1 activation in hippocampal neurons triggers rapid intracellular responses that reduce hyperarousal and improve contextual fear discrimination (35, 77).

Neurodegenerative disorders, particularly Alzheimer’s disease (AD), further substantiate ER-β and GPER1’s neuroprotective roles. Data from Baez-Jurado and Rincón-Benavides (2019) and others reveal that ER-β activation reduces amyloidogenic processing, dampens microglial priming, and improves mitochondrial efficiency—mechanisms that align with inflammation-associated treatment resistance in chronic mood disorders (77–79). This convergence across neuroinflammatory, synaptic, and endocrine axes illustrates the cross-system relevance of estrogenic modulation beyond mood alone.

Notably, clinical trials have demonstrated the psychiatric efficacy of selective estrogen receptor modulators in non-female populations. In schizophrenia, raloxifene reduced negative symptoms and cognitive rigidity in both pre- and post-menopausal women without feminizing side effects or endocrine compromise (27, 80). These findings confirm that centrally selective ER-β and GPER1 modulation retains efficacy across sexes under appropriate pharmacological conditions.

By situating male BD within this shared receptor ecology, the proposed model gains both mechanistic and translational robustness. Rather than representing a sex-specific anomaly, estrogenic neuromodulation reflects a conserved psychiatric mechanism adaptable across disorders. This positions ER-β/GPER1 agonism as a viable intervention not only for BD, but also for schizophrenia, PTSD, and neurodegenerative conditions—particularly within male subgroups marked by chronic inflammation or monoaminergic instability. Future clinical trial designs should therefore explore these receptor axes across diagnostic boundaries using biomarker-guided stratification (27, 36).

### Limitations, ethical considerations, and future directions

While the proposed estradiol–SERM strategy is biologically plausible and mechanistically grounded, several unresolved challenges remain regarding its long-term application in male psychiatric populations. Chief among these is the potential for neuroendocrine disruption due to chronic estrogen receptor modulation. Extended activation of ER-β and GPER1 may induce feedback alterations in the HPG axis, affect androgen–estrogen balance, or generate downstream effects on spermatogenesis, pituitary signaling, and reproductive hormone cascades (7, 24, 81). As such, rigorous endocrine surveillance protocols must be embedded in all clinical trials. These should include serial assessments of serum estradiol, total and free testosterone, SHBG, follicle-stimulating hormone (FSH) and luteinizing hormone (LH), and inflammatory markers such as CRP and IL-6 to evaluate safety, target engagement, and dynamic hormonal shifts (82).

Additionally, therapeutic stigma represents a key ethical and practical challenge. Hormonal interventions, particularly those involving estrogen, may elicit concern or resistance among male participants due to gendered associations with feminization. To mitigate these risks, informed consent processes must be both transparent and demystifying—emphasizing the central nervous system–specific, receptor-targeted intent of estradiol–SERM therapy. Incorporating perspectives from clinicians, ethicists, and patient advocacy groups will be essential in defining tolerable risk-benefit thresholds, refining language around treatment framing, and improving enrollment acceptability (83, 84).

Looking forward, future clinical trials should adopt biomarker-enriched, stratified designs to optimize therapeutic yield and mitigate off-target risk. Recruitment should prioritize individuals with molecular or physiological profiles predictive of estrogenic responsiveness. Candidate stratification variables may include estrogen receptor polymorphisms—such as estrogen receptor 1 (ESR1) rs9340817 and estrogen receptor 2 (ESR2) rs1256049—which have been associated with differential receptor sensitivity, stimulant reactivity, and antidepressant response (73, 85). Dopaminergic tone may serve as another key moderator: for instance, catechol-O-methyltransferase (COMT) Val/Val genotype carriers, known to exhibit prefrontal dopamine hypofunction, may benefit disproportionately from GPER1-mediated catecholamine stabilization (86, 87).

Neurophysiological indices such as quantitative electroencephalography (QEEG) patterns or mismatch negativity (MMN) amplitudes, alongside peripheral markers like BDNF, can further refine subgroup identification—capturing individuals whose underlying pathology reflects synaptic dysregulation, neurotrophic insufficiency, or HPA axis dysfunction (88, 89). By embedding these endophenotypic indicators into trial methodology, estradiol–SERM interventions may be precisely targeted to those most likely to benefit (88–90), enhancing both efficacy and ethical justifiability.

While precise estradiol dosing for male psychiatric populations remains understudied, converging data from related clinical contexts support a plausible physiological range. Trials in women with schizophrenia have employed transdermal 17β-estradiol at 100–200 μg/day, improving mood and cognitive outcomes without significant feminization (67). Preclinical studies in male rodents demonstrate neuromodulatory effects at low estradiol doses, particularly within the 25–100 μg/kg range, with selective ER-β and GPER1 activation (38, 91). A recent meta-analysis confirms that estrogenic modulation within this window can impact dopaminergic, 5-HT1A, and glutamatergic pathways central to neuropsychiatric disorders (92). These findings collectively support the feasibility of initiating low-dose estradiol protocols in sentinel male cohorts, titrated conservatively based on endocrine monitoring, mood stabilization, and cognitive endpoints.

Ultimately, the safe translation of receptor-targeted hormone therapy into male BD care will depend on the convergence of endocrine monitoring, patient-centered trial design, and mechanistically informed biomarker stratification. This paradigm embodies the next step in operationalizing precision psychiatry—not as an aspirational concept but as a practical framework grounded in molecular pathology and therapeutic selectivity (30, 31).

## Conclusion

The clinical impasse posed by TR-BD—particularly in male cohorts—demands a paradigmatic shift away from symptom-targeted polypharmacy toward sex-informed, receptor-specific neuromodulation. This manuscript proposes a mechanistically grounded, dual-axis intervention strategy: the co-administration of low-dose estradiol with SERMs such as raloxifene. This approach is designed to selectively engage central estrogenic receptors—ER-β and GPER1—while antagonizing peripheral ER-α–mediated effects (27, 28). These targets converge on neuroplastic, inflammatory, and monoaminergic pathways implicated in affective destabilization and pharmacoresistance, providing a precision-guided adjunctive framework for male BD care. This therapeutic model is anchored in molecular evidence linking estradiol signaling to neurotrophic induction (e.g., BDNF), inflammatory attenuation (via IL-6 and TNF-α suppression), and HPA axis recalibration—core pathways dysregulated across affective, cognitive, and stress-responsive domains in male BD (9, 32, 35). Rather than repurposing a female-centric endocrinology, this model advances a male-vulnerability hypothesis—one that locates affective lability and treatment refractoriness in sex-specific receptor distributions, dopaminergic instability, and developmental neuroimmune priming.

Importantly, this strategy draws precedent from schizophrenia-spectrum interventions, where SERM–estradiol combinations have improved negative symptoms and cognitive processing without inducing feminizing sequelae (14). The incorporation of a SERM buffer ensures receptor-specific CNS engagement while preserving peripheral hormonal homeostasis—rendering the approach not only mechanistically plausible but clinically viable within male endocrine profiles (27, 28).

The implications are scalable: this receptor-based framework extends naturally to other inflammation-linked, plasticity-deficient conditions, including treatment-resistant depression, schizoaffective disorder, and complex PTSD. In these disorders—where monoaminergic strategies yield diminishing returns—neuroendocrine-circuit targeting may restore regulatory control and affective integration (9, 24).

As precision psychiatry matures into a biologically stratified discipline, the strategic reintroduction of sex hormones as therapeutic agents—selectively channeled, molecularly buffered, and translationally informed—may mark a pivotal advance in male-focused psychiatric innovation (14). Estradiol is not merely an exogenous supplement; it is a potent neuromodulator whose receptor-level dynamics intersect directly with the neuroarchitecture of emotional regulation (9, 36, 93).

Moving forward, early-phase clinical trials in male cohorts must adopt biomarker-enriched designs, integrating cytokine profiling, receptor polymorphism screening, and dynamic hormone assays to validate not only clinical efficacy but also neural target engagement and downstream transcriptional shifts (35, 36, 38). This is not hormonal repurposing—it is a rational reclassification of estradiol as a circuit-specific neuromodulator in male psychiatric care (11).

## Data Availability

The original contributions presented in the study are included in the article/**Supplementary Material**. Further inquiries can be directed to the corresponding author.
