# Supplementary material for: A framework for safe estradiol modulation in male bipolar disorder: theoretical justification for SERM-enabled adjunctive therapy
Source: Front Psychiatry. 2025 Sep 9;16:1644175. doi: 10.3389/fpsyt.2025.1644175 (PMC12454395; doi:10.3389/fpsyt.2025.1644175)
Supplement: Supplementary file 1 [file DataSheet1.docx]

**Appendix A: Detailed Mechanisms of Estrogenic Signaling in Bipolar Disorder**

**Overview of Estrogenic Signaling Pathways**

**Estrogen modulates neural function through two principal mechanisms:**

- Genomic signaling via classical nuclear estrogen receptors (ER-α and ER-β), involving ligand-induced receptor dimerization, nuclear translocation, and transcriptional regulation at EREs on neuroplasticity-related genes such as *BDNF*, *NRG1*, and *ARC* [6, 7].
- Non-genomic signaling via the GPER1/GPR30, which rapidly activates PI3K/Akt, MAPK/ERK, and cAMP/PKA cascades—modulating CREB phosphorylation, synaptic vesicle dynamics, and glutamatergic receptor trafficking [20, 33].

**ER-β: Transcriptional and Neuroimmune Regulation**

ER-β is highly expressed in limbic and prefrontal regions—including the hippocampus, medial prefrontal cortex (mPFC), and amygdala—and exerts the following effects:

- Neurotrophic support via CREB-mediated upregulation of *BDNF* and related genes [18].
- Anti-inflammatory regulation through inhibition of IKK signaling and nuclear NF-κB translocation, thereby downregulating IL-6 and TNF-α production [7, 30].
- Promotion of neuronal resilience under conditions of oxidative and inflammatory stress [21].

**GPER1: Non-Genomic Stress Buffering and Synaptic Plasticity**

GPR30 activation contributes to:

- Rapid, non-genomic modulation of limbic-prefrontal circuits under stress, via MAPK/ERK and cAMP/PKA pathway activation.
- Regulation of glial–neuronal crosstalk, including astrocyte–microglia signaling, influencing neuroinflammatory tone and glial-mediated synaptic protection [26, 31].

**Relevance to Bipolar Disorder (BD) Pathophysiology**

These receptor-mediated pathways converge on domains disrupted in BD, including:

- Impaired neurotrophic signaling
- Chronic low-grade neuroinflammation
- Affective lability and executive dysfunction

Together, ER-β and GPR30 provide mechanistically distinct, druggable targets for receptor-specific neuromodulation—supporting their utility in sex-informed and treatment-resistant populations [29, 32].
